# Improving Resident Physician Participation in Reporting Patient Safety and Quality Concerns

**DOI:** 10.31486/toj.24.0016

**Published:** 2024

**Authors:** Steven R. Craig, Hayden L. Smith, Patrick J. Shaeffer

**Affiliations:** Medical Education Services, UnityPoint Health–Iowa Methodist Medical Center, Des Moines, IA

**Keywords:** *Hospital incident reporting*, *internship and residency*, *patient safety*, *quality improvement*

## Abstract

**Background:** Reporting medical errors, near misses, and adverse events is an important component of improving patient safety and resident learning. Studies have revealed that event reporting rates can be low for physicians, resident physicians, and fellows. The objective of this quality improvement project was to improve resident reporting of patient safety and quality events and engage residents in the analysis of events at a community-based teaching hospital in the United States.

**Methods:** We developed a program to engage 122 residents from 6 Accreditation Council for Graduate Medical Education–accredited residency programs using a multifaceted approach that included instructing residents how to use the hospital's adverse event reporting system; requiring first-year residents to submit at least 1 report; reviewing all resident reports during a monthly multidisciplinary meeting; and ensuring that each resident who submitted a report received feedback on how the concern was being addressed.

**Results:** The program resulted in a 41.8% (95% CI 31%-53%) absolute increase in the number of residents reporting a concern, and resident submissions led to several documented improvements in patient care. A survey was administered to the residents who submitted reports, and the majority (76.0% response rate) expressed satisfaction with both the reporting system and the feedback about how their submission was being addressed. The responding residents agreed that they were more likely to submit reports because of their experience with the program and that they felt the program would improve safety and the quality of care at the institution.

**Conclusion:** This quality improvement project successfully increased resident event reporting and engaged residents in the review of submitted events. The program can serve as a model for other teaching hospitals.

## INTRODUCTION

The Accreditation Council for Graduate Medical Education (ACGME) Common Program Requirements for residency programs specifies that all residents must participate in the reporting of patient safety events: “Residents … must know their responsibilities in reporting patient safety events and unsafe conditions at the clinical site, including how to report such events; and, be provided with summary information of their institution's patient safety reports.”^1^ Further, the ACGME has established patient safety and health care quality as focus areas in the Clinical Learning Environment Review (CLER), with safety event reporting and resident engagement in patient safety event reviews listed as important priorities.^2^

Reporting medical errors, near misses, and adverse events is an important component of improving patient safety. Institutions regularly employ standardized event reporting systems to identify patient safety and quality concerns that require attention, but underreporting of events may hinder the value of these incident reporting systems. A number of studies have revealed that event reporting rates can be very low for physicians and equally sparse for resident physicians and fellows.^3-6^

## METHODS

This quality improvement project consisted of a 2-year program designed to increase resident reporting of patient safety events and engage residents in patient safety analysis at a midwestern university–affiliated, community-based teaching hospital, a 650-bed tertiary academic medical center with 6 sponsored residency programs and 122 trainees.

Prior to the development of the quality improvement project, a baseline resident survey was conducted at the hospital in 2020. Survey results confirmed that few residents (approximately 20%) were using the institution's reporting system to submit patient safety and quality concerns. The 2 primary barriers reported on the survey were a lack of familiarity with how to use the reporting system and a lack of feedback on how submitted concerns were being addressed. Subsequently, a team of 7 resident physicians and 2 faculty mentors designed and initiated a project addressing the 2 primary concerns reported on the baseline resident survey.

During the 2021-2022 academic year, the team developed educational materials on how to submit a patient safety or quality concern using the event reporting system (RLDatix). The educational materials included a 2-minute video and a 1-page handout that were shared with the 122 residents working at the institution.

**Figure 1.**
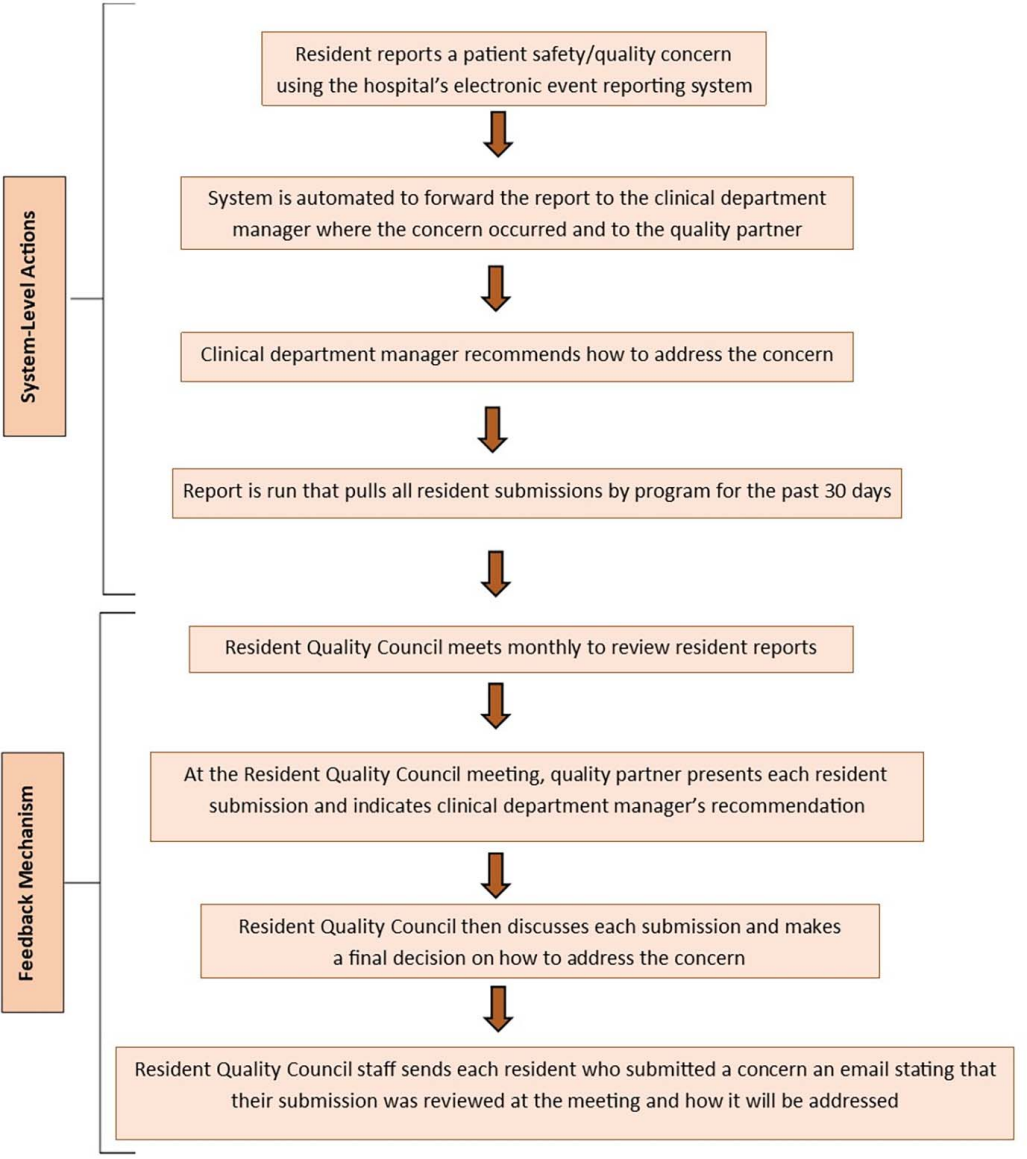
Overview of the patient safety and quality event reporting program. The quality partner is the Quality Improvement department employee who is responsible for the event reporting system.

The team also developed a protocol for presenting all resident submissions at the monthly 1-hour Resident Quality Council meetings and having each submission reviewed by resident representatives from each residency program and representatives from Quality Improvement, Pharmacy, Nursing, the Director of Graduate Medical Education/Designated Institutional Official, and the Vice President of Medical Affairs/Chief Safety Officer. After the Resident Quality Council meeting, all residents who submitted a reviewed concern were sent an email with information on how the submission was or would be addressed. Figure 1 outlines the steps in the protocol.

A new program requirement was added during the 2022-2023 academic year: all first-year residents were required to submit at least 1 patient safety concern during the academic year.

The follow-up email that each submitting resident received asked the resident to complete a brief electronic survey about their satisfaction with the event reporting system. The survey contained 4 structured questions asking residents to indicate their overall satisfaction with the reporting system, satisfaction with the feedback provided by the Resident Quality Council, whether their experience made them more or less likely to report concerns in the future, and whether they thought the reporting system would improve safety and quality of care at the institution. Responses to the questions were on a 5-point Likert scale (ie, strongly agree to strongly disagree; very satisfied to very dissatisfied; much more likely to much less likely). The survey also had an open-ended question soliciting additional feedback about the system and how resident reports were handled. Residents who did not complete the initial survey were sent a reminder email with a link to the survey 7 days after the original email request.

Outcomes examined in this project were the number of event submissions from residents, types of patient safety and quality concerns submitted, and resident satisfaction with both the submission process and follow-up information by the Resident Quality Council. To capture the impact of the complete program, data are presented from the preintervention baseline year (2020-2021) and from the second year postintervention (2022-2023). Review and dissemination of program results received exempt status (File ID 2023-009) from the hospital institutional review board.

## RESULTS

**Figure 2.**
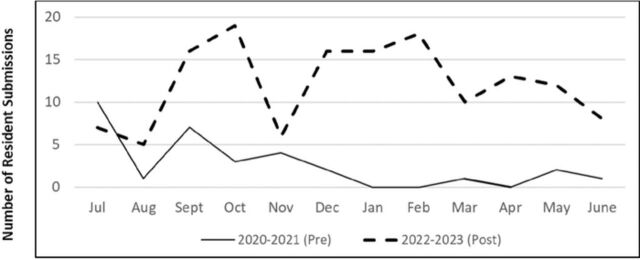
Preintervention (pre) and postintervention (post) counts of resident event report submissions by month. A total of 29 reports were submitted during the preintervention baseline year (2020-2021) vs 136 total reports during the second postintervention year (2022-2023).

The number of event reports for patient safety and quality concerns submitted by residents increased during the second year of the project (Figure 2). During the preintervention baseline academic year (July 2020 to June 2021), 24 of the 122 residents submitted a total of 29 concerns, and 10 of the submissions were from the 39 first-year residents. During year 2 of the study (July 2022 to June 2023), 75 of the 122 residents submitted a total of 136 concerns. The difference in the number of unique individual residents submitting reports across the 2 time periods is a 41.8% (95% CI 31%-53%) absolute increase.

The increase in submissions from first-year residents was expected given the new requirement that they all submit at least 1 report during the year (ie, 10/39 vs 38/39 first-year residents); however, we also saw an increase in reports submitted by upper-level residents (ie, 14/89 vs 37/89 upper-level residents).

**Figure 3.**
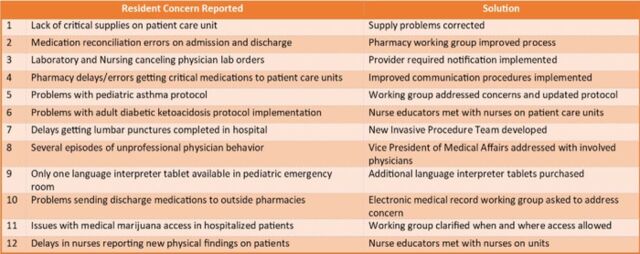
Examples of patient safety and quality concerns submitted by residents during the 2022-2023 academic year.

The patient safety and quality concerns reported by residents included several significant problems that were addressed and led to improvements in care at the institution. Figure 3 presents examples of 12 thematic patient safety and quality concerns submitted by residents in the second year of the project and how the institution addressed the concerns.

Of the 75 residents with an event submission in the second year, 57 completed the program survey (76.0% response rate). The majority of the surveys were positive. Responding residents agreed that the event reporting system allowed them to adequately explain their patient safety and quality concern (82.5% strongly agreed or agreed). Responses also showed a majority of residents were satisfied with the information they received on how their patient safety and quality concern was being addressed (80.7% very satisfied or satisfied), and residents agreed that using the system to report patient safety and quality concerns would improve patient care at the institution (86.0% strongly agreed or agreed). Based on their experience, residents reported they were more likely to use the system to report concerns in the future (68.4% much more likely or more likely). The survey questions and detailed count data for the responses are provided in the Table.

**Table.** Resident Event Reporting System Survey and Responses

Question Number	Survey Question	Response, n=57
1	I feel like the questions from the event reporting system form allowed me to adequately explain my concern.	
	5 = Strongly agree	27 (47.4)
	4 = Agree	20 (35.1)
	3 = Neither agree nor disagree	7 (12.3)
	2 = Disagree	2 (3.5)
	1 = Strongly disagree	1 (1.8)
2	How satisfied are you with the follow-up information you were provided about your submitted concern?	
	5 = Very satisfied	35 (61.4)
	4 = Satisfied	11 (19.3)
	3 = Neither satisfied nor dissatisfied	8 (14.0)
	2 = Dissatisfied	1 (1.8)
	1 = Very dissatisfied	2 (3.5)
3	I think event reporting submissions will improve the safety and quality of care provided at UnityPoint Health-Des Moines.	
	5 = Strongly agree	32 (56.1)
	4 = Agree	17 (29.8)
	3 = Neither agree nor disagree	6 (10.5)
	2 = Disagree	1 (1.8)
	1 = Strongly disagree	1 (1.8)
4	Given your experience with submitting this concern using the event reporting system, are you more or less likely to submit another concern using the system?	
	5 = Much more likely	15 (26.3)
	4 = More likely	24 (42.1)
	3 = Neither more likely nor less likely	16 (28.1)
	2 = Less likely	1 (1.8)
	1 = Much less likely	1 (1.8)
5	Please share any other comments/feedback about the use of the event reporting system to report quality and safety concerns at UnityPoint Health-Des Moines.	
6	Which residency program are you in? Check one:	
	□ Family Medicine □ Internal Medicine □ Pediatrics □ Psychiatry □ Surgery □ Transitional Year	

Note: Data are presented as n (%).

## DISCUSSION

This program at a university-affiliated community-based teaching hospital successfully increased resident use of an event reporting system and could serve as a model for other teaching hospitals.

The patient safety, quality, and medical education literature reports a number of related programs that have been implemented at teaching hospitals. These programs used various strategies to increase resident-identified patient safety and quality concerns and to engage residents in analyzing these events.^6-13^ However, none of the prior studies indicated that all resident event report submissions would be reviewed and that feedback would be sent to the submitting residents on how their concerns would be addressed—a key provision of our program.

The literature also describes efforts to create resident quality and safety councils.^14-17^ Although the focus and design of these councils varies, they are linked by the common effort to involve residents in addressing safety and quality concerns. Our program is unique in that the Resident Quality Council met monthly and reviewed all resident adverse event reports submitted during the prior month. The Resident Quality Council included resident representatives from each of the residency programs who met with hospital quality and patient safety leaders. Leaders at our hospital recognized the value of continuing the program to improve patient safety and address quality concerns and agreed to take responsibility for tracking resident submissions, bringing them for discussion at the monthly Resident Quality Council meetings, and sending follow-up emails to residents about how the submissions are being addressed starting in the 2023-2024 academic year.

Limitations of our program included initial difficulties locating all resident submissions in the event reporting system. The program also required a support person to work closely with the Quality Improvement department to identify and summarize the resident submissions to be discussed at the monthly Resident Quality Council meetings. These tasks may require substantial time commitments. Finally, this program worked well at a teaching hospital with 6 residency programs and 122 total residents. Hospitals with very large graduate medical education footprints may generate significantly more resident-reported concerns and may require adjustments. Of note, although the program provided documentation that safety and quality concerns were being addressed, a formal review of the impact of these changes on future patient outcomes has not been conducted.

## CONCLUSION

Positive results from this quality improvement program included improving resident reporting of patient safety events and quality concerns and involving residents in the review process. Resident submissions identified 12 areas of concern. Resident satisfaction with the program was high, and the program was seen as a positive way to improve the quality and safety of patient care at the hospital.

## Notes

*This article meets the Accreditation Council for Graduate Medical Education and the American Board of Medical Specialties Maintenance of Certification competencies for Patient Care, Medical Knowledge, Systems-Based Practice, and Practice-Based Learning and Improvement.*
